# Face adaptation: Investigating non-configural contrast alterations

**DOI:** 10.3758/s13414-025-03157-9

**Published:** 2025-12-02

**Authors:** Nils Kloeckner, Ronja Mueller, Marie Buerling, Claus-Christian Carbon, Tilo Strobach

**Affiliations:** 1 Department of Psychology, MSH Medical School Hamburg, Am Kaiserkai 1, 20457 Hamburg, Germany; 2 Bamberg Graduate School of Affective and Cognitive Sciences (BaGrACS), University of Bamberg, Bamberg, Germany; 3 ICAN Institute for Cognitive and Affective Neuroscience, MSH Medical School Hamburg, Hamburg, Germany; 4 IFPM Institute for Forensic Psychology and Forensic Medicine, MSH Medical School Hamburg, Hamburg, Germany; 5 Department of General Psychology and Methodology, University of Bamberg, Bamberg, Germany; 6 Research Group EPÆG (Ergonomics, Psychological Æsthetics, Gestalt), Bamberg, Germany

**Keywords:** Face adaptation, Face perception, Face memory, Non-configural face information, Contrast information

## Abstract

The process of adapting facial representations plays a critical role in face perception and memory, representing an interplay of bottom-up and top-down mechanisms. This process allows individuals to recognize faces despite dynamic changes, for example, aging. However, a full understanding of the adaptation characteristics of non-configural facial information is still lacking in the face-processing literature. The present study investigates face aftereffects in response to facial contrast information, extending the research beyond recent studies on adaptation regarding brightness and color saturation information to a new non-configural facial information type. The research involved four experiments using celebrity face images manipulated for facial contrast, with intervals ranging from 300 ms (Experiment 1) to 5 min (Experiment 2) between adaptation and test phases. Experiment 3 used inverted adaptation faces to investigate whether adaptation effects transfer to upright test faces. The results demonstrate adaptation effects for facial contrast that are robust over time and do not transfer from inverted to upright faces. In addition, these effect sizes were compared to those of brightness and saturation information (Experiment 4), revealing no significant differences in magnitude. In general, the present findings suggest that non-configural facial contrast information is an integral part of face representations, representing an interplay of bottom-up and top-down mechanisms in face processing. All data are available on the Open Science Framework.

## Introduction

Human faces are of significant importance when recognizing individuals and navigating social interactions. They have a dynamic character resulting from changes in, among others, emotions, health, aging, visual conditions, and context. However, despite these dynamic changes, most people are able to recognize faces with high accuracy, particularly if we are familiar with them. We can achieve this accuracy by constantly updating our mental representations of faces – adapting them to the dynamics of our environment. However, when observing manipulated faces, this adaptation process can cause a strong misperception in the faces that are subsequently perceived – a phenomenon called “face aftereffect” (e.g., Webster & Maclin, 1999). This misperception can reveal whether specific face information is part of the mental face representation and, therefore, relevant for face identification. The present study investigates this adaptation effect for the first time using non-configural facial contrast information. The results are discussed in the context of recently published research on other non-configural information such as saturation, brightness, complexion, or freckles (Mueller et al., 2021a, 2021b; Utz et al., 2023a, 2023b).

### Previous studies on face adaptation

The pioneering work of Webster and Maclin (1999) was one of the first to systematically demonstrate visual adaptation effects in response to manipulations in human faces. In their adaptation study, participants were exposed to heavily distorted faces during an initial adaptation phase. The images were distorted by either expanding or contracting the frontal-view image of the face relative to a midpoint on the nose. In a subsequent test phase, the authors observed a pronounced bias in face perception, where the veridical (i.e., original), unaltered image was perceived as slightly distorted in the opposite direction to the adapting stimulus. However, images manipulated in the direction of the adaptation image were perceived as the original image. Since this pioneering research, numerous studies on face adaptation have been conducted, revealing various face-adaptation effects (for an overview, see Strobach & Carbon, 2013). Research exploring different operational parameters, for instance, by varying the interval between the adaptation and test phases, ranging from milliseconds to hours and even days, has shown that face-adaptation effects are remarkably robust over time (Carbon & Ditye, 2011). These effects can even transfer to different faces, as demonstrated by studies in which stimulus similarity was varied between the adaptation and test phases using the same image in both phases, different images of the same person, or images of other persons, resembling the transfer levels pictorial, structural, and cross-identity, respectively (Carbon & Ditye, 2011; Carbon et al., 2007). These findings on the time robustness and the transferability of face-adaptation effects suggest that these effects are better explained by changes at the level of memory representations rather than by changes occurring solely at a perceptual or retinal level (Carbon & Ditye, 2012; Strobach et al., 2011; Webster & Macleod, 2011).

### Previous studies on adaptation with non-configural facial information

Most previous studies have primarily focused on adaptation effects related to configural face information, which involves second-order spatial relations (e.g., eye distance, eyes-mouth distance; Maurer et al., 2002; Piepers & Robbins, 2012; for a review, see Strobach & Carbon, 2013). However, there is only a limited amount of adaptation research on other types of face information, although face recognition relies on a broader range of facial information beyond just configural information, such as non-configural information (e.g., Cabeza & Kato, 2000; Macho & Leder, 1998; Mondloch et al., 2002; Rakover & Teucher, 1997). The limited exploration of adaptation effects in non-configural face information in prior studies inspired the research of Mueller and Utz and their respective colleagues (Mueller et al., 2021a, 2021b; Utz et al., 2023a, 2023b). These studies represent pioneering work in demonstrating that manipulations in non-configural face attributes, such as brightness and saturation, produce enduring and transferable adaptation effects. In particular, Mueller et al. (2021b) employed celebrity faces with brightness manipulations, either extremely decreased or increased, as adaptation stimuli. The subsequent test phase featured pairs of slightly manipulated face images (either decreased or increased brightness) alongside non-manipulated images. Participants had to select the veridical image from pairs of a manipulated and a non-manipulated image. By more frequently selecting the test image that most closely resembled the adaptor they had previously seen, participants demonstrated adaptation effects in their image selection. These adaptation effects were observed for all three transfer levels (i.e., pictorial, structural, and cross-identity); however, it was less pronounced for the structural and cross-identity transfer levels than for the pictorial one. These effects on all three transfer levels suggest that the adaptation of non-configural information in face representations is not only limited to the presented identity but also affects superordinate concepts encompassing various identities that share common underlying face structures. The adaptation effects were exclusively related to upright faces and could not be induced with scrambled or inverted faces. In general, this set of findings highlights the significance of non-configural brightness information in face representations during the encoding and retention of faces.

In their subsequent study, Mueller et al. (2021a) showed adaptation effects on saturation information (another non-configural face information) at all transfer levels. However, the results on saturation adaptation slightly differed from the adaptation effects on brightness information: the authors found an adaptation effect for saturation information only when faces with strongly increased saturation were employed as adaptors. Further, adaptation effects for both types of investigated non-configural face information (i.e., brightness and saturation information) were shown to be differently robust in time. Whereas adaptation effects on both types of information persisted for 300 ms and 3,000 ms, only the brightness adaptation effect was able to endure for 5 min. In general, more recent studies extend the above-mentioned adaptation effects to other naturally occurring non-configural changes, such as changes in skin tone due to sun tanning (Utz et al., 2023a) and intensity of freckles (Utz et al., 2023b).

In addition to the different types of non-configural face information mentioned above, faces also contain other non-configural information that has not yet been considered in adaptation research. One of these, so far largely uninvestigated, non-configural information is contrast. In order to further investigate the significance of non-configural facial features for face representation, contrast should also be examined more closely. Unlike others (e.g., Russell, 2009; Russell et al., 2016), who understand facial contrast as the luminance contrast between internal facial features and the surrounding skin, we use facial contrast according to Webster and Macleod (2011) as physical contrast of the face image (e.g., how faded or distinct the image is) in the present study. Since contrast has the ability to modulate the neural response, and, moreover, it is essential for encoding (e.g., Russell, 2009), contrast is potentially relevant for understanding adaptation effects.

It has been shown that facial contrast decreases with age and serves as a cue for age perception (Porcheron et al., 2013, 2017). Modifying facial contrast also affects the perception of health (Russell et al., 2016) and beauty or facial attractiveness (Russell, 2003; Störmer & Alvarez, 2016). Face recognition is strongly disrupted by inverting image contrast (Galper, 1970). Because this manipulation preserves all of the spatial information in the image, such a contrast inversion effect demonstrates that face recognition not only depends on a generic representation of spatial information, but contrast information may also seem to play a vital role (Webster & Maclin, 1999). In addition to brightness and color saturation, facial contrast thus provides essential information about a face. It can, therefore, be assumed that facial contrast is used in face recognition too and therefore should be contained in mental representations of faces.

Unlike the perception of absolute brightness or color saturation, contrast perception is about perceiving the difference in brightness and saturation at the visual edge between two objects or an object and its surroundings. Hence, the perception of contrast is the perception of edges between two neighboring surfaces in the visual field (for an overview, see Kesserwani, 2020). Visual edges are one of the most informative features of the face, as they determine the extent and position of various objects in the respective image. Given the importance of edge detection, it is not surprising that our visual system is particularly specialized in detecting edges (for information on lateral inhibition as the neuronal basis for contrast enhancement see, e.g., Ratliff, 1972). Nevertheless, compared to cells in primary visual cortex, higher visual areas that encode objects and faces are less affected by variations in contrast (Avidan et al., 2002; Rolls & Baylis, 1986). On the other hand, strong transfer across contrast was demonstrated for high-level shape aftereffects (Anderson et al., 2007; Suzuki, 2001), which is compatible with the adaptation in higher visual areas (Webster & Macleod, 2011). Because of these characteristics of contrast perception, the mere fact that brightness and saturation are types of non-configural face information that demonstrate an adaptation effect and are thus stored in memory does not mean that the same is valid for contrast.

In addition, previous studies on brightness and saturation have produced slightly different results (Mueller et al., 2021a, 2021b). For example, saturation is less or hardly robust over time, but brightness is robust for at least 5 min (and maybe even 20 min; see Mueller et al., 2021b). These findings suggest that adaptation effects vary for different types of non-configural information, making it difficult to infer how adaptation effects manifest for contrast based on our previous studies. While the information on brightness and saturation is more transient (e.g., a face’s brightness can be manipulated by variations in illumination; a face’s saturation can change with the person’s emotional or health state), the information on facial contrast seems to be much more stable over time. It could therefore be a facial feature that can uniquely define a face (e.g., age and facial attractiveness). Configural (i.e., spatial) facial information can also be considered highly stable, with significant changes occurring primarily during childhood and adolescence. Compared to non-configural facial information examined so far (particularly saturation), adaptation effects to configural facial information appear to be considerably more robust, persisting for at least 1 week (see Carbon & Ditye, 2011). One hypothesis is that the robustness of these adaptation effects may be related to the variability of facial information in daily life (see, e.g., Mueller et al., 2021a). Since contrast information is likely more stable than brightness or saturation information, adaptation effects to contrast may also exhibit greater robustness compared to effects on brightness and saturation. An investigation of the retention of contrast information in face memory and the comparison with brightness and color saturation could be of interest.

### The present study

The present study extends the exploration of adaptation effects associated with non-configural contrast information. Five main objectives will be addressed: (1) determining if adaptation to contrast manipulations (both increased and decreased) occur; (2) assessing the robustness of contrast adaptation effects over time; (3) assessing the transfer of contrast adaptation effects to images that share common underlying face structures; (4) investigating whether these contrast adaptation effects are specific to upright faces after an inverted adaptor; and (5) comparing the magnitude of adaptation effects between contrast, saturation, and brightness information.

Four experiments were conducted in the present study, all basically following a procedure similar to Mueller et al. (2021a) with a focus on contrast manipulations. All of these experiments used celebrity images as adaptation and test stimuli, manipulated in contrast. In Experiments 1, 3, and 4, the interval between the adaptation phase and the test phase was relatively short (i.e., 300 ms). By extending this interval to 5 min in Experiment 2, we aimed to explore the temporal robustness of adaptation effects, gaining insights into the processing level of contrast adaptation. This can help to determine if these effects are exclusively confined to perceptual and retinal processing or extend to higher levels of the visual pathway, potentially influencing face memory representations. Experiments 1, 2, and 3 incorporated three transfer levels: pictorial, structural, and cross-identity, allowing us to examine the representational levels at which adaptation effects might occur. To directly compare adaptation effects on contrast with those on saturation and brightness, Experiment 4 manipulated the contrast, saturation, and brightness of the adaptation and test stimuli. In this latter experiment, only the pictorial transfer level was used to create a feasible experimental design.

In all experiments, participants were either exposed to adaptation stimuli with (1) significant decreases in contrast (or saturation/brightness in Experiment 4), (2) no contrast (or saturation/brightness in Experiment 4) manipulation, or (3) significant increases in contrast (or saturation/brightness in Experiment 4), depending on their assigned experimental groups. A two-alternative forced-choice (2AFC) test followed, instructing participants to select the veridical image out of two image versions: one displaying a non-manipulated image and the other showing a slightly manipulated image in terms of contrast (or saturation/brightness in Experiment 4), either decreased or increased. If adaptation generally occurs, we hypothesize that the strong manipulations seen during the adaptation phase would be integrated into participants’ stored face representations, leading to alterations towards the adaptor. Consequently, non-manipulated images would appear manipulated in the opposite direction to the adaptor (e.g., after adapting to an image with increased contrast, a non-manipulated image would appear to have decreased contrast, and vice versa). Thus, during the 2AFC test, participants were expected to select the face version more similar to the adaptor, as this image version corresponds most to the updated face representation in memory.

## Experiment 1

In the present study, we aimed to investigate properties of mental representations of faces by examining face-adaptation effects for non-configural contrast information. Individuals are equipped with those mental representations when faces are well known. To tackle mental representations of well-known faces, we present images of celebrities who are familiar to the participants. In Experiment 1, the interval between the adaptation and test stimuli was set at a relatively short duration of 300 ms. As outlined above, our hypothesis predicted that participants would demonstrate a distinct bias in selecting images more closely to the previously observed adaptor during a subsequent test phase.

### Methods

#### Participants

Forty-eight undergraduate students from the Medical School Hamburg (30 females, 18 males; *M*_age_ = 23.3 years, range 18–47 years) participated in Experiment 1. Previous studies have demonstrated large adaptation effects for both brightness and saturation (Mueller et al., 2021a, 2021b). Therefore, the required sample size of *N* = 48 was determined a priori with G*Power (Faul et al., 2007), considering a mixed-design analysis of variance (ANOVA) with a 3 (between-subjects) × 3 (within-subjects) factorial design, capable of detecting a large effect size *f* of 0.4 (Cohen, 1988), with a significance level *α* of.05, and a test power (1 − β) of 0.85. Participation in the study was either rewarded with €20 or credit points as part of course requirements. All participants were unaware of the experiment’s objective.

The participants’ vision abilities were evaluated using the Freiburg Visual Acuity and Contrast Test (Bach, 1996). Additionally, to detect any color perception anomalies, a brief version of the Ishihara color test (Ishihara, 1917) was administered. Only individuals with normal or corrected-to-normal vision, as determined by these tests, participated in the subsequent testing. Participants were randomly assigned to one of three groups, each exposed to different adaptation stimuli: decreased contrast, non-manipulated contrast, or increased contrast.

All participants provided informed written consent before their involvement in the study. Ethical approval for the research was obtained from the ethics board of the Medical School Hamburg on 9 March 2017, and the study adhered to the guidelines of the Declaration of Helsinki.

#### Apparatus and stimuli

To construct a set of familiar celebrities, the methods of the present experiment closely followed the methods used by Mueller et al., (2021a, 2021b). As reported in these papers, a pool of 70 celebrity names was randomly gathered from newspaper articles and through input from colleagues. Subsequently, a survey involving 92 participants (66 females; *M*_age_ 21.7 years, range 18–30 years) was conducted, where the participants were instructed to assess the familiarity of each celebrity on a 5-point Likert scale (1 = *unfamiliar*; 5 = *very familiar*) and provide the names of the celebrities if known. Based on the survey results, the 30 celebrities^1^ with the highest familiarity ratings and most frequently named were chosen as stimulus material for the subsequent tests.

For each of the 30 celebrities, two distinct images (A and B), which were different from those used in the survey, were selected. These images fulfilled specific criteria, displaying the celebrity's full face in a frontal view, with a direct gaze, no eyeglasses, and no hair obscuring any facial features (e.g., eyes, nose, or mouth). Additionally, the chosen images were of high resolution. The 30 celebrities were randomly assigned to three different celebrity groups.

**Fig. 1:**
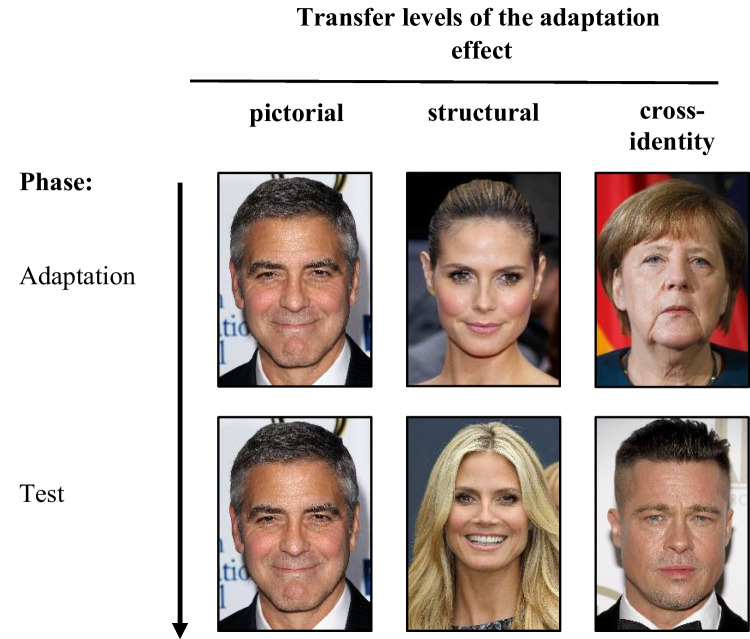
Illustration of the three transfer levels pictorial, structural, and cross-identity between adaptation and test phase in Experiments 1–3 (only pictorial in Experiment 4). It is important to note that these images were not utilized in the original study but are provided solely for illustrative purposes. The figure is sourced from Mueller et al. (2021b), and appropriate permissions and image licenses have been secured from the respective copyright holders (sources: ©Drop of Light/Shutterstock.com, Tinseltown/Shutterstock.com, s_bukley/Shutterstock.com)

Since two images (A and B) were available for each celebrity, there were six unique stimulus sets. These sets were utilized to create three different transfer levels: pictorial, structural, and cross-identity. These levels varied in terms of the shared information between images presented during the adaptation and test phases (see Fig. 1). In the pictorial transfer level, the test condition involves presenting pictorially identical images between the adaptation and test phases (e.g., George Clooney as both the adaptor and the test stimulus). The structural transfer level entails using different images of the same identity for the adaptation and test phases (e.g., image A of Heidi Klum as the adaptor stimulus and image B of Heidi Klum as the test stimulus). On the cross-identity transfer level, the images used in the adaptation and test phases display different identities (e.g., an image of Angela Merkel as the adaptor and an image of Brad Pitt as the test stimulus).

**Fig. 2:**
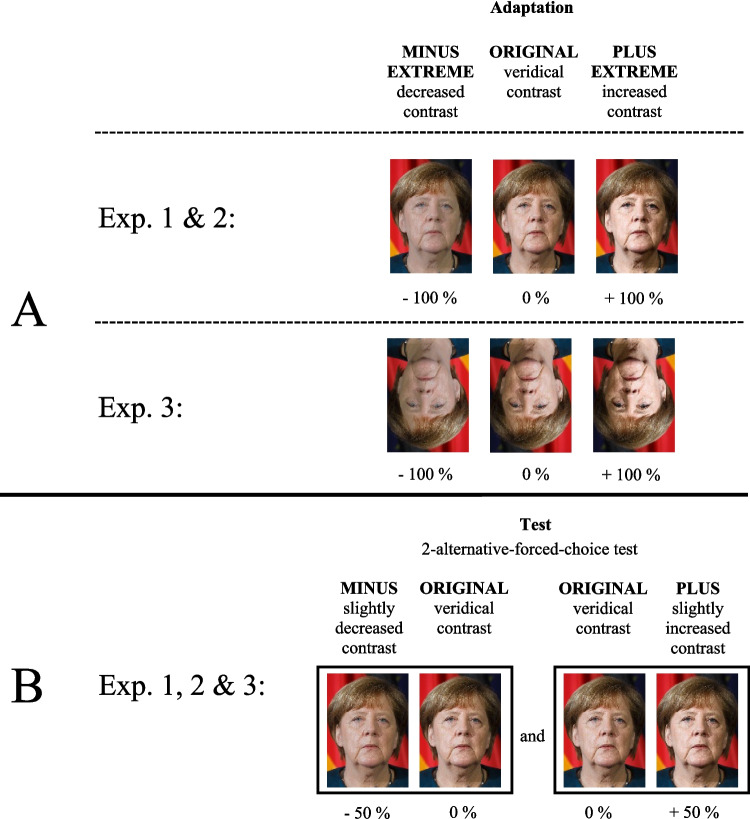
Illustration of the different image versions in Experiments 1–4 using Angela Merkel’s face as an example. (**A**) The different adaptor versions employed in Experiments 1–3. Notably, Experiments 1 and 2 utilized identical adaptation images, while Experiment 3 involved inverted versions of the adaptor images utilized in Experiments 1 and 2. (**B**) The image versions during the test phase in Experiments 1–3, utilizing a two-alternative forced-choice (2AFC) test approach. In this test, two images were presented, with one being the original image and the other either decreased or increased in contrast. The position of the images (centered left or centered right) was alternated during the test phase. The depicted images of Angela Merkel were not utilized in the original study but are provided solely for illustrative purposes. All necessary permissions and image licenses have been obtained from the respective copyright holders (source: ©Drop of Light/Shutterstock.com)

All images underwent manipulation by altering the facial contrast of the presented faces (excluding hair) using Adobe Photoshop CC (Version 19.0). In this program, using the contrast, a slider increases or decreases the overall range of tonal values in the image. If the contrast is increased or decreased in this way, the color saturation is always increased or decreased at least slightly by default. Different degrees of contrast manipulation were applied, resulting in five different image versions by adjusting Photoshop’s contrast control on − 100%, − 50%, 0%, + 50%, and + 100%, as illustrated in Fig. 2. Note that the manipulation levels applied in this study differ from those used by Mueller and colleagues (2021a, 2021b). After piloting revealed that the manipulation strengths used by Mueller and colleagues were perceived as less intense when applied to contrast, the authors decided to intensify the manipulation of contrast. The image size was approximately 330× 412 pixels, which corresponds approximately to the dimensions 8.8 × 10.9 cm at a pixel density of approximately 96 PPI (pixels per inch).

Depending on which group participants were assigned to (between-participants factor), they were presented with different adaptation stimuli. The groups included those who saw an unaltered contrast image (0%, ORIGINAL), an image with strongly decreased contrast (− 100%, MINUS EXTREME), or an image with strongly increased contrast (+ 100%, PLUS EXTREME) as an adaptor. It is worth noting that the MINUS EXTREME and PLUS EXTREME adaptation stimuli were clearly recognizable as manipulations.

Participants were presented with two versions of each image during the test phase. One version displayed the ORIGINAL image (i.e., we did not alter this image in terms of contrast), while the other version exhibited a slightly manipulated version with either − 50% contrast (MINUS) or + 50% contrast (PLUS). It is important to acknowledge that any potential manipulations were not immediately obvious, ensuring that the face images sufficiently represented our accumulated familiarity with the presented identities. The experiment was designed using Experiment Builder 2.2.1 (SR Research) and conducted on Lenovo PCs with 23-in. computer screens, operating at a resolution of 1,920 × 1,080 pixels.

#### Procedure

**Fig. 3:**
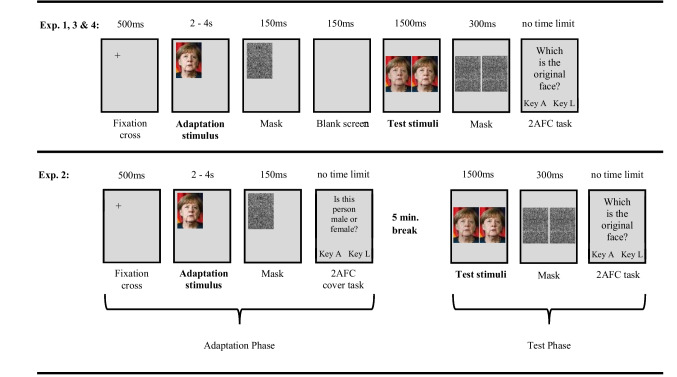
Schematic illustration of the trial structure in Experiments 1–4. Trials in Experiments 1, 3, and 4 share identical timing parameters but differ in their stimulus material (see Fig. 1 for stimulus details). In Experiment 2, we employed a block-wise procedure, where the adaptation and test phase are presented in two different blocks, separated by a 5-min break (see text for details). In Experiment 3, inverted adaptors were employed. In Experiment 4, not only contrast manipulations (as illustrated) were used, but also saturation and brightness manipulations (see Fig. 5 for details). Please note that the adaptation stimuli were always extremely manipulated, whereas the test stimuli are either original or slightly modified faces. An illustrative image of Angela Merkel was used for demonstration purposes. The figure’s content has been adapted from Mueller et al. (2021b), and all required permissions and image licenses have been obtained from the respective copyright holders (Sources: © Drop of Light/Shutterstock.com)

As depicted in Fig. 3, each trial encompassed both the adaptation and the test phases. Every trial started with the presentation of a fixation cross at the center of the subsequent stimulus position for a duration of 500 ms. Following this, the adaptor image was displayed, featuring either the ORIGINAL image or one of the two extreme manipulations (MINUS EXTREME or PLUS EXTREME), based on the participant’s group assignment (as illustrated in Fig. 2).

To avoid adaption to specific screen locations, the adaptor images were presented in one of six different screen positions (top-left, top-center, top-right, bottom-left, bottom-center, or bottom-right). Additionally, to introduce task variability and minimize fatigue without reducing the inspection time of the adaptation stimuli, we displayed the adaptor images for durations of 2,000, 3,000 or 4,000 ms. Each adaptor image was presented four times at each presentation duration and twice in each screen position, resulting in a total of 12 presentations for each individual adaptor throughout the entire experiment.

During the adaptation phase, participants encountered only two (out of six) stimulus sets of celebrity faces, which were selected from two different celebrity groups (e.g., image set A or B). The third group of celebrity faces was not displayed during the adaptation phase. The specific stimulus sets presented to each participant were determined in advance, and the order of stimulus presentations was randomized within the experiment. The allocation of presented stimulus sets was balanced among participants within each adaptation group. Subsequent to the adaptor presentation, in each trial, participants were briefly exposed to a 150-ms backward mask to eliminate possible afterimages (Turvey, 1973). After the mask, a blank screen appeared for 150 ms, resulting in an interstimulus interval of 300 ms between the adaptation and test stimuli.

After the blank screen presentation, participants were prompted to engage in the 2AFC task, in which they viewed two versions of the same image that only differed in contrast. These images, comprising the ORIGINAL image paired with either a MINUS image version (− 50% contrast) or a PLUS image version (+ 50% contrast), were displayed for 1,500 ms. The position of both presented images (centered left or centered right) was randomized and balanced throughout the experiment.

During the adaptation phase, only two celebrity groups were presented, but during the test phase, all three groups were shown to encompass all three transfer levels. Depending on the transfer level, different stimulus sets were presented during the adaptation and test phases. The use of the stimulus sets could, for example, be as follows:

1. Pictorial: Trials presenting images of image set A from celebrity group 1 were used as both adaptors and test stimuli.
2. Structural: Trials displayed image set A from celebrity group 2 during adaptation, and image set B of the same celebrity group served as test stimuli.
3. Cross-identity: Trials presented half of image set A from celebrity group 1 and half of image set A of group 2 during adaptation, and image set A from celebrity group 3 was used as test stimuli.

Several test versions were created to ensure that, across test versions/participants, the use of stimulus sets and groups was balanced within the respective transfer conditions. The adaptation phase of the cross-identity transfer level was always formed using half of the adaptor set of the pictorial transfer level and half of the adaptor set of the structural transfer level. The contrast of the adaptors was maintained constant within each participant group across all transfer levels. The image sets presented during the adaptation and test phase were predetermined and balanced across participants, just like the trial order, which was randomized within the experiment.

After displaying the two test images, a backward mask was shown for 300 ms to eliminate any afterimages and prevent prolonged exposure to the test stimuli. Subsequently, participants were instructed to select the test image they believed to be the original face, using a specific button on the keyboard corresponding to the image position (key “A” for images presented on the left side, key “L” for images on the right side). Following the procedure of Carbon et al. (2007), participants were told to base their decision on their existing knowledge about the celebrity, such as images encountered in the media. By doing so, participants were encouraged to access the mental representation of the celebrity stored in memory when identifying the “veridical” image during the test phase. Furthermore, since the previously displayed adaptation stimuli were evidently strongly manipulated, participants were explicitly informed not to base their decision on these adaptor images when selecting the non-manipulated image in the test phase.

The experiment comprised a total of 360 trials. After completing half of the trials (i.e., 180 trials), a break was provided to allow participants to relax and inform them that they had completed half of the experiment. Participants independently initiated the second half. After completing this part of the experiment, the participants were once again presented with the previously shown celebrity images. This time, their task was to assess the familiarity of each celebrity, with instructions to indicate whether they were familiar with the celebrity from the media. Participants responded with either “yes” or “no” by pressing the keys “A” or “L,” respectively. The ratings obtained from this familiarity judgment were utilized to exclude trials featuring celebrities who were unknown to the participants. This step was necessary as adaptation effects on face representations are expected to be more pronounced for familiar faces, given that familiar faces are already well represented in memory (for a comparison of adaptation effects on familiar vs. unfamiliar faces, see the study by Hills & Lewis, 2012). Overall, the experiment had a duration of approximately 60 min.

### Results and discussion

On average, 96.2% of the face stimuli received familiarity ratings (individually ranging from 70 to 100%) and were subsequently included in further analysis. All individual outliers, which were defined as response times exceeding 3 standard deviations (*SD*s) above the individual mean response time, were removed. Additionally, trials with response times faster than 200 ms were considered anticipatory responses and were also removed. As a consequence, a total of 20.5% of all trials in this experiment were excluded from the analysis; 14.3% of all trials due to anticipatory responses.

The main dependent variable of interest was the average test face selection in the 2AFC task. This variable served as an indicator of whether prior exposure to strongly manipulated images, for instance, caused a shift in face perception. The variable was scored based on the degree of manipulation of the selected test images: A score of −50 was assigned to the MINUS images, a score of 0 to the ORIGINAL images, and a score of + 50 to the PLUS images.^2^ Based on the experimental design, a two-way, mixed-design ANOVA was performed, with the between-participants factor being the adaptation group (MINUS EXTREME, ORIGINAL, and PLUS EXTREME) and the within-participants factor being the transfer level (pictorial, structural, and cross-identity). For non-significant effects, we report Bayes factors expressed as BF_01_, grading the intensity of the evidence that the data provide for H_0_ versus H_1_ (for details, see Wagenmakers et al., 2018).^3^

**Fig. 4:**
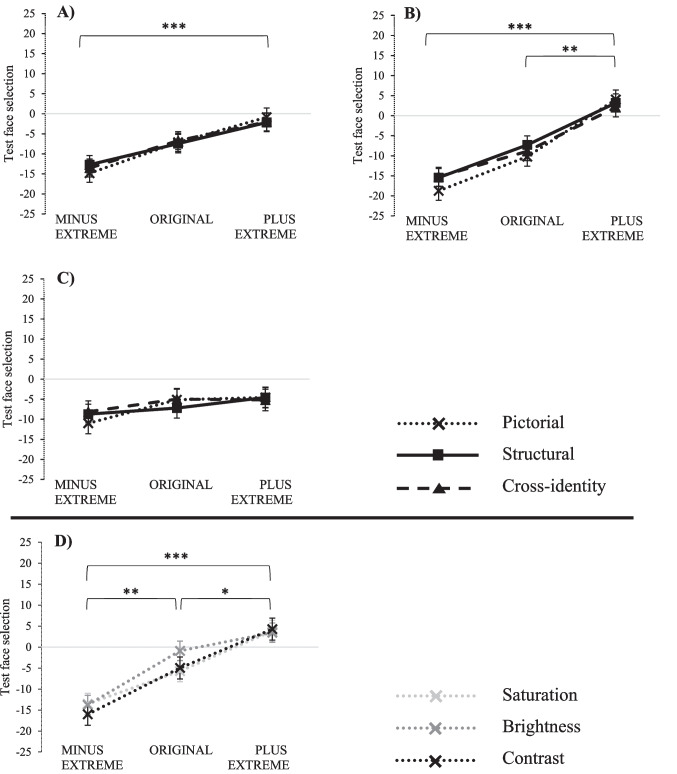
Illustration of the results of the two-alternative forced-choice (2AFC) task on the factors adaptation group and transfer level for Experiments 1–4. The vertical axis represents the average level of contrast (panels A–C for Exps. 1–3) or of contrast, saturation, and brightness (panel D for Exp. 4) of the chosen test images in the 2AFC format (see text for detailed information regarding the determination of the dependent variable). The error bars indicate a range of ± 1 standard error of the mean. Please note that in panels A–C lines represent different transfer levels, whereas in panel D lines represent different non-configural information types

As illustrated in Fig. 4A, there was a main effect of adaptation group, *F*(2, 45) = 7.67, *p* =.001, ${\upeta }_{p}^{2}$ =.25, with the means as follows: *M*_MINUS EXTREME_ =  − 13.70 (*SD* = 5.73), *M*_ORIGINAL_ =  − 7.10 (*SD* = 9.53), and *M*_PLUS EXTREME_ =  − 1.69 (*SD* = 10.13). Bonferroni-corrected comparisons showed significant differences between MINUS EXTREME and PLUS EXTREME: *p* <.001, Cohen’s *d* =  − 1.46. However, there were no significant differences between MINUS EXTREME and ORIGINAL: *p* =.112, *d* =  − 0.84, and between ORIGINAL and PLUS EXTREME: *p* =.254, *d* =  − 0.55. These results suggest that the MINUS EXTREME and PLUS EXTREME adaptation groups showed a noticeable bias towards different directions (one for decreased contrast and the other for increased contrast), indicating an adaptation effect of facial contrast. On the other hand, the main effect of transfer level was not significant, *F*(2, 90) < 1, *BF*_01_ = 14.19, and there was no interaction between transfer level and adaptation group, *F*(4, 90) = 1.264, *p* =.29, ${\upeta }_{p}^{2}$ =.053, *BF*_01_ = 4.22. These findings suggest that the observed adaptation effect not only exists on an image-specific level but also transfers to other images or even different identities. It is somewhat surprising that we did not observe a significant modulation of the adaptation effect across transfer levels in this experiment, as previous studies using similar paradigms and short intervals have found such transfer patterns, for instance in adaptation to facial brightness (e.g., Mueller et al., 2021b). One possible explanation for the absence of a significant interaction is that, although the adaptation effect reached significance and was strong, it was less pronounced than in the previous study investigating face-adaptation effects on brightness information. This reduced effect size may have limited the ability to detect differences across transfer levels.

## Experiment 2

Based on the findings of Experiment 1, which demonstrated face-adaptation effects on contrast alterations, Experiment 2 aimed to further investigate the temporal robustness of these effects by increasing the time interval between the adaptation and test phases from 300 ms to 5 min. This delay, based on the idea of Carbon et al. (2007), was previously demonstrated to be sensitive in revealing differences in the stability of adaptation effects for brightness versus saturation manipulations, with only the former showing robust effects over time (Mueller et al., 2021a, 2021b).

A central question addressed in Experiment 2 is, therefore, whether contrast adaptation effects persist over longer time intervals and remain stable even in the presence of interfering visual input in the form of additional face stimuli. While previous studies have shown that adaptation to configural face features can last for several minutes or even hours and days (Carbon & Ditye, 2012; Carbon et al., 2007), evidence for similarly robust effects for non-configural features remains limited. If adaptation effects are still present after such a delay and in the context of interference, this would support the notion that these effects are not limited to early visual or retinal processes, but may reflect changes at the level of higher-level cognition or memory representations.

### Methods

#### Participants

Forty-eight undergraduate students from the Medical School Hamburg (38 females, ten males; *M*_age_ = 23.4 years, range 19–40 years) were tested individually. The sample size, study requirements, and conditions for this experiment were consistent with the previous experiment. None of the participants had taken part in the prior experiment.

#### Apparatus and stimuli

The apparatus and stimuli employed in this experiment were identical to those in Experiment 1.

#### Procedure

In contrast to the previous experiment, Experiment 2 incorporated a separation between the adaptation and test phases. As depicted in Fig. 3, adaptation phase trials were initiated with the presentation of a fixation cross for 500 ms, centered within the subsequent stimulus position. Participants were then exposed to the adaptor, which varied depending on their group assignment: an ORIGINAL image or one of the two extreme versions (MINUS EXTREME or PLUS EXTREME), displayed for 2,000–4,000 ms. The adaptors were presented in one of six distinct screen positions, with these parameters (presentation time and position) being balanced across trials.

Following adaptor exposure, a backward mask appeared for 150 ms, succeeded by a screen displaying a gender discrimination task (asking in German: “Is this person a woman or a man?”) as a cover task. Participants indicated their choice by pressing the “A” key for a man or the “L” key for a woman. As in the preceding experiment, the image sets presented during the adaptation phase were balanced across participants. After the adaptation phase, a 5-min break was introduced, during which participants were presented with a geographical text to prevent the mental recall of previously viewed images.

Following this break, the test phase was administered. To encompass all three transfer levels (pictorial, structural, and cross-identity) in this phase, all three celebrity groups were presented during the test phase. Image sets employed for the test phase were balanced across participants. Test stimuli were displayed for 1,500 ms, and backward masks followed each test-stimulus presentation. The position of test stimuli was randomized and balanced across trials. Subsequently, participants were tasked with selecting the veridical image from the two previously presented options (the 2AFC task), indicated by pressing either the “A” or “L” key. The experiment comprised 360 adaptation and 120 test trials and had a total duration of approximately 50 min. Similar to Experiment 1, participants engaged in a familiarity rating task after both the adaptation and test phases.

### Results and discussion

On average, 97.4% of the face stimuli were rated as familiar, with individual ratings ranging from 57 to 100%. Only trials presenting celebrities familiar to the participants were included in further analysis. The outlier analyses and 2AFC analysis followed a similar approach to the previous experiment. A total of 4.3% of all trials in this experiment were excluded from the analysis, with no anticipatory responses observed. (Note that this result of the exclusion procedure clearly differs from the other experiments. In these experiments, the proportion of excluded anticipatory responses was obviously higher than in the present experiment. These differences might result from the different trial structures, where the other experiments included both adaptation and test phases within each trial, whereas adaptation and test phases were separated in the present experiment.)

We conducted a two-way, mixed-design ANOVA with the between-participants factor adaptation group (MINUS EXTREME, ORIGINAL, and PLUS EXTREME) and the within-participants factor transfer level (pictorial, structural, and cross-identity). As shown in Fig. 4B, the analysis revealed a significant main effect of adaptation group, *F*(2, 45) = 20.10, *p* <.001, ${\upeta }_{p}^{2}$ =.472, with the means as follows: *M*_MINUS EXTREME_ =  − 16.48 (*SD* = 5.72), *M*_ORIGINAL_ =  − 8.80 (*SD* = 6.89), and *M*_PLUS EXTREME_ = 3.09 (*SD* = 12.33). Bonferroni-corrected comparisons showed significant differences between MINUS EXTREME and PLUS EXTREME: *p* <.001, *d* =  − 2.04, and between ORIGINAL and PLUS EXTREME: *p* =.001, *d* =  − 1.19. The difference between MINUS EXTREME and ORIGINAL just missed statistical significance: *p* =.052, *d* =  − 1.21. Although Experiments 1 and 2 both revealed adaptation effects, we acknowledge that the difference in paradigm structure (trial-by-trial vs. blocked) is confounded with the manipulation of delay duration. This limits the direct comparability of the two experiments. We therefore interpret their results independently. Nevertheless, the results of Experiment 2 indicate that adaptation effects on contrast are temporally robust to last at least 5 min.

The main effect of transfer level was not significant, *F*(2, 90) = 2.70, *p* =.073, ${\upeta }_{p}^{2}$ =.057, *BF*_01_ = 1.79. There was a significant interaction between transfer level and adaptation group, *F*(4, 90) = 2.56, *p* =.044, ${\upeta }_{p}^{2}$ =.102. The mean difference between PLUS EXTREME and MINUS EXTREME was larger for the pictorial transfer level (*M* = 22.78) than for the structural transfer level (*M* = 18.64) and the cross-identity transfer level (*M* = 17.30), respectively. This suggests that the observed adaptation effect was strongest for the pictorial transfer level. Due to the blocked structure of Experiment 2, participants were exposed to a broad set of adapting faces before the test phase. Thus, any observed adaptation effects likely reflect the combined influence of all adaptation stimuli rather than specific adaptor–test relations, limiting clear conclusions about transfer levels (pictorial, structural, cross-identity). This limitation, however, is not unique to the blocked design. In paradigms without blocking (e.g., Experiment 1), participants might also adapt to multiple faces across trials, which may similarly lead to cumulative effects. Thus, both designs are subject to the broader challenge of isolating transfer effects in the context of multiple adaptors, and any interpretation regarding transfer level must be made with this constraint in mind.

Importantly, the persistence of adaptation effects after a 5-min delay suggests that the effects are not limited to short-lived perceptual aftereffects. Rather, they appear to reflect more robust representational changes. This interpretation aligns with previous findings on configural face adaptation, which show effects lasting minutes to days (Carbon & Ditye, 2012; Carbon et al., 2007), and complements earlier work on non-configural features such as brightness and saturation, where similar effects have been observed (Mueller et al., 2021a, 2021b). Thus, the present findings provide further evidence for the temporal robustness of adaptation effects for facial contrast and support the view that these effects may involve higher-level memory-based representations rather than purely low-level visual mechanisms.

To address the possibility that the adaptation effects found are based on recency effects (i.e., a tendency to better remember or be more influenced by information that appeared most recently), we conducted an additional analysis. Given that recency effects typically decrease over time (Glanzer & Cunitz, 1966), we divided the test phase of Experiment 2 in halves to examine the time course of the general adaptation effects across all transfer conditions. We conducted a mixed-design ANOVA with the between-participants factor adaptation group (MINUS EXTREME, ORIGINAL, and PLUS EXTREME) and the within-participants factor test half (first, second); however, we did not include a transfer level factor and aggregated over the different transfer levels. We observed a strong main effect of adaptation group, *F*(2, 45) = 20.53, *p* <.001, ${\upeta }_{p}^{2}$=.477, suggesting an overall adaptation effect regardless of test half. However, there was no interaction effect between test halves and adaptation groups, *F*(2, 90) < 1, *p* =.920, ${\upeta }_{p}^{2}$=.004, *BF*_01_ = 5.93, indicating that adaptation effects did not diminish over time. These findings reinforce the assumption that the adaptation effects are not based on recency effects. Interestingly, a main effect for test half emerged, *F*(1, 45) = 9.37, *p* =.004, ${\upeta }_{p}^{2}$=.172, with the mean response in the first half (*M* =  − 2.905) differing significantly from that in the second half (*M* =  − 4.437). This result indicates that across all adaptation groups, the second half of the test phase yielded significantly more negative scores than the first half, suggesting a notably stronger negativity bias in the latter half of the experiment (for more details on the negativity bias, see also the *General discussion*).

## Experiment 3

To investigate whether the effects observed in Experiments 1 and 2 are specific to upright faces after an inverted adaptor, we conducted the following experiment using inverted face stimuli that were altered in contrast during the adaptation phase. Inverted face stimuli share similarities with upright images, except for their orientation. Despite these similarities, face inversion leads to significant recognition impairments, making inverted faces very suitable as a form of “non-face stimulus” (for a review, see Valentine, 1988; see also, e.g., Yin, 1969). If Experiment 3 reveals no adaptation effects or adaptation effects smaller than in Experiments 1 and 2, this would indicate that the adaptation mechanism is sensitive to face orientation.

### Methods

The methods of Experiment 3 are identical to the methods of Experiment 1 despite the following changes. Forty-eight undergraduate students from the Medical School Hamburg (46 females, two males; *M*_age_ = 23.4 years, range 18–58 years) participated in Experiment 3. None of the participants in this experiment had taken part in Experiments 1 or 2 previously. The adaptation stimuli (but not the test stimuli) used in this experiment were inverted versions of the stimuli presented in Experiment 1 (see Fig. 1). Although the adaptor stimuli in Experiment 3 differed from the test images in their orientation, we will continue using the term “pictorial” for the sake of consistency with the descriptions of the other experiments. In the context of Experiment 3, “pictorial” refers to the pictorial congruence of the basic image.

### Results and discussion

Due to a technical issue, the familiarity ratings of 26 participants were not recorded in this experiment. For this reason, celebrities rated as unfamiliar could not be excluded from the main analysis, and we analyzed the data of all 48 participants, regardless of the familiarity ratings. The outlier analyses and 2AFC analysis followed a similar approach to Experiment 1. A total of 12.2% of all trials in this experiment had to be excluded from the analysis (all excluded trials were anticipatory responses).

A two-way, mixed-design ANOVA was conducted with the between-participants factor adaptation group (MINUS EXTREME, ORIGINAL, and PLUS EXTREME) and the within-participants factor transfer level (pictorial, structural, and cross-identity). As shown in Fig. 4C, the main effect for the adaptation group was not significant, *F*(2, 45) =.94, *p* =.399, ${\upeta }_{p}^{2}$ =.040, *BF*_01_ = 1.69. Thus, we did not find any evidence of a general adaptation effect regarding contrast alterations of inverted faces. In addition, neither the main effect of transfer level, *F*(2, 90) = 1.41, *p* =.249, ${\upeta }_{p}^{2}$=.030, *BF*_01_ = 4.81, nor the interaction between transfer level and adaptation group, *F*(4, 90) = 2.10, *p* =.087, ${\upeta }_{p}^{2}$ =.085, *BF*_01_ = 1.67, reached significance.^4^

Our results showed no significant adaptation effect in any of the tested transfer levels when the adaptor was inverted. This suggests that face adaptation for contrast information does not transfer from inverted to upright faces, indicating that the adaptation mechanism is sensitive to face orientation. However, it is important to note that this finding does not in itself demonstrate face-specificity in a general sense. The lack of transfer might be explained by low-level visual differences introduced by inversion, such as changes in the retinal configuration of brightness, saturation, or contrast patterns. Thus, our results indicate that face adaptation for contrast information is specific to the upright configuration of faces, but not necessarily that the effect is exclusive to faces as a category.

## Experiment 4

To compare the strength of the adaptation effects for several non-configural face information, we conducted Experiment 4, in which face-adaptation effects for different non-configural information types (i.e., contrast, saturation, and brightness information) were examined. To do so, participants were again randomly assigned to one of three adaptation groups (i.e., MINUS EXTREME, ORIGINAL, and PLUS EXTREME) as in Experiments 1–3. Additionally, participants were presented with alterations of all three non-configural information types in a within-participants design.

### Methods

**Fig. 5:**
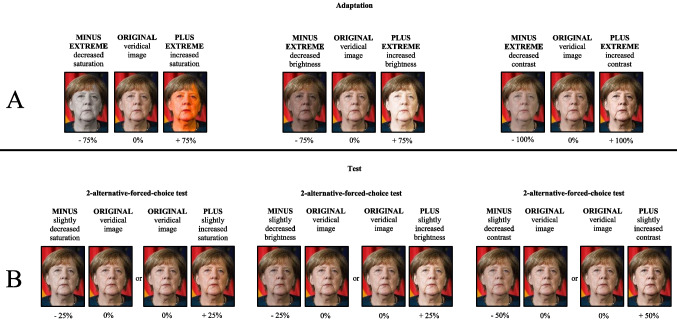
The different image versions from Experiment 4 are illustrated by the face of Angela Merkel. (**A**) Illustration of the different adaptor versions. (**B**) Illustration of the image versions during the test phase. Within the two-alternative forced-choice (2AFC) test, two images are displayed (the original image together with either an image with decreased saturation/brightness/contrast or an image with increased saturation/brightness/contrast)

The methods of Experiment 4 are identical to the methods of Experiment 1, despite the following changes: 48 undergraduate students from the Medical School Hamburg (29 females, 19 males; *M*_age_ = 24 years, range 18–34 years) participated in Experiment 4. None of the participants in this experiment had taken part in the previous experiments. Each non-configural information type (i.e., contrast, saturation, and brightness) was presented in a separate block of 120 trials, resulting in a total of 360 trials. The manipulation levels for contrast were identical to those of the previous experiments, while the manipulations for saturation and brightness were adopted from Mueller and colleagues (2021a, 2021b). In the adaptation phase, saturation and brightness were reduced or increased by 75%. In the test phase, however, saturation and brightness were reduced or increased by 25% (see Fig. 5). Although the same stimulus sets were used as in the previous experiments, only the pictorial transfer level was employed.

### Results and discussion

On average, 86% of the face stimuli were rated as familiar, with individual ratings ranging from 73 to 100%, and only these stimuli were included in further analysis. The outlier and 2AFC analyses followed a similar approach to the previous experiments. A total of 19.1% of all trials in this experiment were excluded from the analysis, 15.0% of all trials due to anticipatory responses.

Regardless of the different levels of manipulation for contrast on the one hand and saturation and brightness on the other hand, a score of − 50 was assigned to the MINUS images, a score of 0 to the ORIGINAL images, and a score of + 50 to the PLUS images.

A two-way, mixed-design ANOVA was conducted with the between-participants factor adaptation group (MINUS EXTREME, ORIGINAL, and PLUS EXTREME) and the within-participants factor non-configural information type (contrast, saturation, and brightness). As illustrated in Fig. 4D, the main effect for the adaptation group was significant, *F*(2, 45) = 17.86, *p* <.001, ${\upeta }_{p}^{2}$=.443, with the means as follows: *M*_MINUS EXTREME_ =  − 14.45 (*SD* = 7.47), *M*_ORIGINAL_ =  − 3.87 (*SD* = 4.78), and *M*_PLUS EXTREME_ = 3.85 (*SD* = 12.18). Bonferroni-corrected comparisons showed significant differences between MINUS EXTREME and ORIGINAL: *p* =.004, *d* =  − 1.69, between MINUS EXTREME and PLUS EXTREME: *p* <.001, *d* =  − 1.81, and between ORIGINAL and PLUS EXTREME: *p* =.047, *d* =  −.84. Univariate tests for simple effects of adaptation group for each type of non-configural information revealed similar effect sizes: ${\upeta }_{p}^{2}$=.34 for brightness, ${\upeta }_{p}^{2}$=.40 for saturation, and ${\upeta }_{p}^{2}$=.40 for contrast. The main effect of non-configural information type was not significant, *F*(2, 90) = 1.20, *p* =.306, ${\upeta }_{p}^{2}$ =.026, *BF*_01_ = 5.32. The interaction between adaptation group and non-configural information type was also not significant, *F*(4, 90) = 1.33, *p* =.265, ${\upeta }_{p}^{2}$ =.056, *BF*_01_ = 4.36. Thus, we did find strong evidence for face-adaptation effects regardless of non-configural information type with no differences in adaptation strength.

## General discussion

Employing face-adaptation experiments is a valuable method to explore how we perceive faces and store them in memory in a combination of bottom-up and top-down mechanisms (e.g., Carbon & Ditye, 2011). Earlier studies mainly focused on the adaptation of configural aspects of faces, whereas more recently, there has been a shift towards investigating non-configural facial information such as brightness, saturation, freckles, and complexion (Mueller et al., 2021a, 2021b; Utz et al., 2023a, 2023b). However, in this series of adaptation experiments investigating non-configural facial information, the aspect of contrast was still missing.

Although contrast perception plays a crucial role in the perception of objects in general and faces in particular (e.g., Galper, 1970; Porcheron et al., 2013, 2017; Russell, 2003; Russell et al., 2016; Störmer & Alvarez, 2016), it has not been yet investigated whether contrast is also part of the face representation. Therefore, the experiments described here aim to expand the range of face-adaptation studies by investigating if face adaptation to contrast alterations (both increased and decreased) occurs. In case adaptation effects occur, subsequent experiments should assess the temporal robustness of these effects as well as their specificity to faces. Finally, we wanted to compare the magnitude of adaptation effects between contrast alterations and alterations of other non-configural facial features (e.g., saturation and brightness).

### Existence of adaptation effects on contrast alterations and their face specificity

The results of Experiments 1 and 2 clearly show that adaptation effects occur for alterations in facial contrast. The exposure to the applied alterations in contrast causes a clear bias in the perception of subsequent faces. There is a significant, medium-sized main effect for the adaptation group. Original faces are subsequently perceived as shifted away from the adaptor, while there is an increase in the likelihood that slightly manipulated faces (in the direction of the adaptor) would be perceived as the veridical version. The PLUS EXTREME adaptation group shows a significant difference to the MINUS EXTREME adaptation group (Experiments 1 and 2). In addition, there was also a significant difference between the PLUS EXTREME adaptation group and the ORIGINAL condition in Experiment 2.

To determine if the adaptation effect for contrast alterations of Experiments 1 and 2 is specific for upright faces, we employed inverted face stimuli as adaptors in Experiment 3. Since Experiment 3 did not show significant adaptation effects, we conclude that the effects observed in Experiments 1 and 2 do not transfer from inverted to upright faces. At least, the adaptation effects include components of adaptation that operate in a way that is sensitive to face orientation. Importantly, this does not allow us to draw strong conclusions about face specificity per se. The inversion of the adaptor alters the retinal configuration of spatial and color features, which may prevent transfer even if similar processing mechanisms are involved. Therefore, while our data show that face-adaptation effects for contrast information are tuned to the upright orientation of faces, they do not rule out the possibility that similar effects could be found for other categories or configurations.

However, the interpretation of the face-adaptation effect for contrast information being face-specific could be further supported by the findings of Experiment 2, where the strength of the adaptation effect systematically decreased with decreasing structural similarity between adaptor and test faces. Adaptation effects were strongest when adaptor and test images were identical (pictorial level), and diminished when only the structural configuration or identity was shared. This graded pattern suggests that the adaptation mechanism operates on representations that reflect face-specific structural properties, rather than purely low-level image features. Taken together, although the results of Experiment 3 do not allow for strong claims regarding face-specificity per se, the findings from Experiment 2 provide converging evidence that the observed contrast adaptation effects may at least partially rely on face-specific mechanisms.

Next to these conclusions regarding the existence of the adaptation effects on facial contrast information, one interesting aspect of our data requires a more elaborate discussion. As in Mueller et al., (2021a, 2021b), results hint at a potential shift in the average selection of all participant groups toward the negative range for all experiments (i.e., a negativity bias). This negativity bias is basically illustrated in all panels of Fig. 4 of the present study. While screen characteristics can, in principle, influence visual perception, we consider it unlikely that display parameters (e.g., brightness or contrast settings) account for the consistent bias toward selecting images with reduced facial contrast. Given that we anticipated a value of around zero for the average selection of the ORIGINAL group across all transfer levels, we systematically analyzed this negativity bias. Therefore, we conducted one-sample t-tests on the mean of this condition for each experiment. The differences between this mean and zero were statistically significant in all individual experiments (Exp. 1: *M* =  − 7.10, *t*(15) =  − 2.98, *p* <.01, *d* =  −.75; Exp. 2: *M* =  − 8.80, *t*(15) =  − 5.11, *p* <.001, *d* =  − 1.28; Exp. 3: *M* =  − 5.91, *t*(15) =  − 2.20, *p* =.044, *d* =  −.55; Exp. 4: *M* =  − 3.87, *t*(15) =  − 3.24, *p* <.01, *d* =  −.81). This consistent finding across experiments indicates that participants seeing non-manipulated upright or inverted face images as the adaptor (ORIGINAL) somehow tend to choose images decreased in contrast (and also brightness and saturation in Experiment 4) as the veridical image in the test phase. Prior research suggests that increases in contrast are perceptually more salient than decreases (Theeuwes, 1992), making images with contrast-enhanced faces more likely to be perceived as artificially altered. In contrast, slight reductions in contrast may go unnoticed and appear more natural. As a result of this asymmetric perceptual sensitivity to increases versus decreases in contrast, participants may be more likely to reject enhanced (PLUS) images as artificially altered, while reduced (MINUS) versions are more likely to be accepted as natural or unmodified (our analyses in Footnote 5 do support this explanation of the negativity bias across the experiments^5^). Furthermore, our data indicate that participants – particularly in Experiments 1, 2, and 3 – had difficulty distinguishing between MINUS and ORIGINAL stimuli, suggesting that slight reductions in contrast may not have been perceptually salient. This likely contributed to the observed negativity bias, as the reduced-contrast version may have been incorrectly perceived as the unaltered, veridical image. Finally, strategic decision heuristics (e.g., selecting the less “edited”-looking image) may have contributed to the consistent preference for lower-contrast faces. Interestingly, the negativity in test face selections increased from the first to the second half of Experiment 2, suggesting a notably stronger negativity bias in the latter half of the experiment. Future research should therefore look into this bias more elaboratively and tests its underlying mechanisms.

### Processing level of adaptation effects on contrast alterations

By demonstrating contrast adaptation effects even when the interval between adaptation and test was extended to 5 min in Experiment 2, the present study provides further evidence that these effects are not limited to low-level retinal or sensory processes. Rather, they likely reflect changes at higher cognitive levels. The use of a block-wise procedure, in which adaptation and test phases were clearly separated, potentially extended the effect duration to up to 50 min – the approximate total length of the experiment. The present findings thus extend prior work on the temporal properties of face adaptation by showing that adaptation effects for facial contrast information is robust over time. Together with previous evidence on configural and non-configural features, this supports the interpretation that adaptation can lead to durable changes in internal representations rather than transient perceptual shifts. In particular, the fact that effects persisted despite multiple intervening face stimuli and a prolonged delay period strengthens the assumption for a representational account of contrast adaptation. It further distinguishes these effects from short-term priming or low-level visual mechanisms.

Furthermore, the observed adaption effects cannot be solely attributed to recency effects. Distracting tasks reduce the recency effects, as previous research has shown (Glanzer & Cunitz, 1966). Therefore, at least in Experiment 2, recency effects were rendered ineffective due to the introduction of a distractor task involving the reading of a geographical text. Furthermore, because of the significant differences between the adaptation and test stimuli, the effects on the cross-identity level (and to some extent even on the structural level) rather contradict a recency effect. In an additional analysis, we divided the test phase of Experiment 2 (i.e., the 5-min experiment) in half to examine general adaptation effects across all transfer conditions. A strong main effect of the adaptation group was observed, suggesting an overall adaptation effect regardless of test half. However, there was no interaction effect between test halves and adaptation groups, indicating that adaptation effects did not diminish over time. Given that recency effects typically decrease over time (Glanzer & Cunitz, 1966), these findings argue against a recency-based explanation for the observed adaptation effects.

### Comparison of the magnitude of adaptation effects between different non-configural facial features

To compare the strength of adaptation effects between contrast alterations and alterations of other non-configural facial information (e.g., saturation and brightness), we conducted Experiment 4. For all three non-configurative facial information types, we found strong adaptation effects that did not differ in magnitude. In addition, contrary to results from experiments on color saturation alone (Mueller et al., 2021a), where an adaptation effect was shown for the increase in color saturation only, Experiment 4 of the present study showed adaptation effects for both increased and decreased saturation information, with no difference in magnitude compared to the adaptation effects on brightness and contrast information.

### Limitations and future research

This study has several methodological and analysis-related caveats that need to be discussed. First, while our paradigm did not include an explicit baseline condition (i.e., we did not assess the face representations of all participants before the start of the experiments), we believe the consistent pattern of results across Experiments 1, 2, and 4 provides strong evidence for an adaptation-based account. That is, in each of these experiments, we observed substantial contrast aftereffects that are robust over time and that closely align with our hypotheses and replicate findings from previous studies deploying the same paradigm, examining different facial features, such as brightness and color saturation (e.g., Mueller et al., 2021a, 2021b). The replication of this pattern both within and across studies makes it unlikely that the effects observed are solely due to individual differences in baseline contrast representations or low-level priming effects. Instead, our data suggest that participants’ internal memory representations were altered, supporting the idea of a genuine adaptation process. Priming, in contrast, is typically understood as the temporary activation of an existing representation, often reflected in faster response times rather than systematic perceptual shifts. Thus, the observed pattern – particularly its persistence and consistency – is more consistent with representational change due to adaptation than with priming (see Mueller et al., 2020, for a comprehensive review on this distinction). In addition, the absence of effects for inverted faces in Experiment 3 provides further evidence for an adaptation-based mechanism. Nonetheless, we acknowledge that including a no-adaptation baseline condition would allow for more precise estimation of the adaptation effect magnitude and would strengthen the causal interpretation in future studies.

Second, due to technical issues, some of the familiarity ratings in Experiment 3 are missing. However, because we obtained consistent results across the main analysis and two follow-up analyses, we assume that our conclusions are justified. Third, in Experiment 3, the adaptor and test stimulus in the “pictorial” transfer condition are not in fact pictorially identical, as they differ in their orientation. Thus, one might expect smaller adaptation effects in the pictorial transfer condition of Experiment 3 compared to Experiment 1, simply because the basic image of the adapter and the test stimulus are still pictorially congruent, but do not have the same orientation. However, the fact that we also found no significant adaptation effects for the structural and cross-identity transfer conditions suggests that the inverted adaptors were not processed in exactly the same way as the upright adaptors. Moreover, because we conducted the power analysis to determine the sample size for detecting strong adaptation effects as we usually observe them with upright adaptors, we can reliably exclude only strong effects in the case of non-significant results. In summary, based on our non-significant results for all three transfer levels, we can conclude that face-adaptation effects for contrast information are specific to upright faces. Fourth, in all four experiments we observed an interesting negativity bias (i.e., a shift in the average selection toward the negative range) that has also been found in previous studies investigating the non-configural facial features brightness and color saturation (Mueller et al., 2021a, 2021b). Across all three transfer conditions, participants in the ORIGINAL adaptation group more frequently selected the image with lower contrast (or brightness or color saturation in Exp. 4) when tasked with choosing the non-manipulated of the two test images. One possible explanation for this bias is that participants’ internal representations were already slightly lower in contrast than the original faces in the images selected by the authors. This highlights the potential role of pre-existing representational differences and underscores the value of including a no-adaptation baseline condition to disentangle such effects from genuine adaptation. Future research should further investigate this negativity bias and also clarify why it apparently becomes stronger the longer the experiment lasts.

### Conclusions

Taken together, the reported experiments clearly provide evidence for strong and robust face-adaptation effects for non-configural contrast information. Since we were able to show that the effects are temporally robust (at least for 5 min), transferable, and specific to (upright) faces, we conclude that the results are better explained by changes at the level of memory representations rather than changes occurring solely at a perceptual or retinal level, supporting the assumption of an interplay of bottom-up and top-down mechanisms. Thus, we were able to demonstrate that in addition to brightness and color saturation, non-configural facial contrast information is an integral part of face representations and can therefore be applied in the process of face recognition.

## Data Availability

All data are available on the Open Science Framework at the following URL: https://osf.io/49tpz/?view_only=c4767b160e6045538b763fcce10a777e.

## Footnotes

1 Musicians (Adele, Amy Winehouse, Ed Sheeran, Justin Bieber, Justin Timberlake, Katy Perry, Pink, Rihanna, and Selena Gomez), actors (Cameron Diaz, Daniel Radcliffe, Elyas M’Barek, Emma Watson, George Clooney, Jennifer Aniston, Leonardo DiCaprio, Til Schweiger, and Will Smith), politicians/head of states (Angela Merkel, Barack Obama, Donald Trump, Queen Elizabeth, Vladimir Putin), athletes (Boris Becker, Cristiano Ronaldo, and Joachim Löw), models (Heidi Klum and Paris Hilton), and entertainers (Thomas Gottschalk and Dieter Bohlen).

2 The largest possible adaptation effect towards decreased contrast would be achieved if a participant consistently selected the MINUS image (scored as − 50) in trials where MINUS and ORIGINAL images were presented in the 2AFC test and always chose the ORIGINAL image (scored as 0) in trials with ORIGINAL and PLUS images. Conversely, the largest adaptation effect towards increased contrast would be attained if a participant consistently selected the PLUS image (scored as + 50) as the veridical one in trials displaying PLUS and ORIGINAL images and always chose the ORIGINAL image (scored as 0) in trials featuring ORIGINAL and MINUS images. Since the different displays (MINUS/ORIGINAL and ORIGINAL/PLUS) of the 2AFC test trials were balanced throughout the experiment, the dependent variable (test face selection) could maximally vary from − 25 to + 25 for each participant and condition.

3 A widely used classification scheme for the interpretation of Bayes factors BF_01_ is that of Jeffreys (1961).

4 In addition to the main analysis, we conducted two follow-up analyses. In a first follow-up analysis, we analyzed the results obtained from all participants and excluded the trials of those participants, where the familiarity ratings were available and considered as unfamiliar celebrities. From the participants with familiarity rating (22 participants), on average, 97.4% of the face stimuli were rated as familiar, with individual ratings ranging from 83 to 100%. The outlier analyses and 2AFC analysis followed a similar approach to Experiment 1 and were conducted for the total number of participants. As in the main analysis, none of the effects reached significance. The main effects for the adaptation group was not significant, *F*(2, 45) =.92, *p* =.406, ${\upeta }_{p}^{2}$ =.039, *BF*_01_ = 1.67. The main effect for transfer level was also not significant, *F*(2, 90) = 1.41, *p* =.249, ${\upeta }_{p}^{2}$=.030, *BF*_01_ = 4.88. Finally, the interaction between transfer level and adaptation group did not reach significance, *F*(4, 90) = 2.34, *p* =.061, ${\upeta }_{p}^{2}$ =.094, *BF*_01_ = 1.08. In a second follow-up analysis, the results were exclusively obtained for the 22 participants from whom the familiarity ratings were recorded (MINUS EXTREME: n = 6, ORIGINAL: n = 9, PLUS EXTREME: n = 7). The main effects for the adaptation group, *F*(2, 19) = 1.09, *p* =.358, ${\upeta }_{p}^{2}$ =.103, *BF*_01_ = 1.26, and for transfer level were not significant, *F*(2, 38) = 0.57, *p* =.570, ${\upeta }_{p}^{2}$=.029, *BF*_01_ = 4.60. The interaction between adaptation group and transfer level was also not significant, *F*(4, 38) = 0.84, *p* =.512, ${\upeta }_{p}^{2}$=.081, *BF*_01_ = 4.27. Thus, similar to the main analysis, these follow-up analyses showed no convincing evidence for adaptation effects with inverted adaptor faces, supporting the assumption of face-adaptation effects in the previous experiments being sensitive to face orientation.

5 Averaged across all subjects in the ORIGINAL adaptation group, we calculated the average rates for selecting the non-manipulated test image for each of our experiments. We did this on the one hand for trials in which the non-manipulated image and the image slightly decreased in contrast (or brightness or saturation in Exp. 4) were presented in the test phase (MINUS) and on the other hand for trials in which the non-manipulated image and the image slightly increased in contrast (or brightness or saturation in Exp. 4) were presented in the test phase (PLUS). The results we obtained for Experiments 1–3 were as follows: Exp. 1: MINUS =.49, PLUS =.77; Exp. 2: MINUS =.44, PLUS =.79; Exp.3: MINUS =.51, PLUS =.73. Although participants did not reject all PLUS versions (what would be indicated by PLUS = 1), these results suggest that they had difficulty in distinguishing the difference between the non-manipulated image and the slightly decreased version. For Exp. 4 we calculated these rates separately for each type of non-configural information: saturation: MINUS =.84, PLUS =.88; brightness: MINUS =.64, PLUS =.89; contrast: MINUS =.67, PLUS =.91. This suggests that in Exp. 4 participants had less difficulty distinguishing between the non-manipulated image and the slightly decreased version.

## Code availability

Code will be made available from the authors upon request.
